# Supplementary material for: Identification of photoperiod-induced specific miRNAs in the adrenal glands of Sunite sheep (Ovis aries)
Source: Front Vet Sci. 2022 Jul 22;9:888207. doi: 10.3389/fvets.2022.888207 (PMC9354845; doi:10.3389/fvets.2022.888207)
Supplement: Supplementary file 1 [file Data_Sheet_1.ZIP › Supplementary file/miRDeep2 file.zip!/miRDeep2 file/Know_Structure/SP42A3/oar-mir-30b.pdf]

miRBase precursor : oar-mir-30b
Total read count : 34236
oar-miR-30b read count : 34033
remaining reads : 203

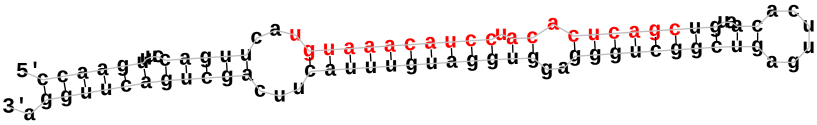


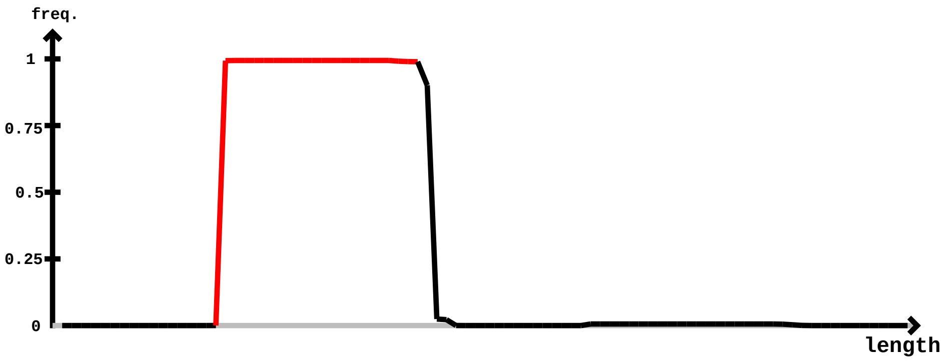


oar-miR-30b

```
5'- ccaaguuuccaguucauguaaacauccuacacucagcuguaacacuugagucggcugggagguggauguuuacuucagcugacuugga   -3'    exp
    ((((((...(((((...((((((((((.((.((((((((..((......))))))))))..)))))))))))...))))))))))).    reads    mm    sample
    ...............auguaaacauccuacacucagcu..................................................    7        0     seq
    ................uguaaacauccuacacuc......................................................    72       0     seq
    ................uguGaacauccuacacuc......................................................    1        1     seq
    ................uguaaacaCccuacacuc......................................................    1        1     seq
    ................uguaaacauccuacacuU......................................................    22       1     seq
    ................uguaaacauccuacacuca.....................................................    50       0     seq
    ................uguaaacauccuacacucag....................................................    17       0     seq
    ................uguaaacauccuacacucagc...................................................    3024     0     seq
    ................uguaaacauccuacacucagcu..................................................    29976    0     seq
    ................uguaaacauccuacacucagcug.................................................    77       0     seq
    ................uguaaacauccuacacucagcugu................................................    754      0     seq
    ................uguaaacauccuacacucagcugua...............................................    6        0     seq
    .................guaaacauccuacacucagcu..................................................    22       0     seq
    .................guaaacauccuacacucagcugu................................................    1        0     seq
    ..................uaaacauccuacacucagcu..................................................    1        0     seq
    ....................aCcauccuacacucagcu..................................................    1        1     seq
    ....................aacauccuacacucagcu..................................................    1        0     seq
    ......................................................cugggagguggauguuu.................    1        0     seq
    ......................................................cugggagguggauguuuU................    1        1     seq
    ......................................................cugggagguggauguuuacu..............    23       0     seq
    ......................................................cugggagguggauguuuacuu.............    77       0     seq
    ......................................................cugggagguggauguuuacuuc............    79       0     seq
    ......................................................cugggagguggauguuuacuuca...........    21       0     seq
    .......................................................ugggagguggauguuuacuuc............    1        0     seq
```
